# Big data and predictive analytics in healthcare in Bangladesh: regulatory challenges

**DOI:** 10.1016/j.heliyon.2021.e07179

**Published:** 2021-05-29

**Authors:** Shafiqul Hassan, Mohsin Dhali, Fazluz Zaman, Muhammad Tanveer

**Affiliations:** a College of Law, Prince Sultan University, Prince Nasser Bin Farhan St, Salah Ad Din, Riyadh 12435, Saudi Arabia; b Department of Business and Law, Federation University Australia, 154-158 Sussex St, Sydney NSW 2000, Australia; c Prince Sultan University, Prince Nasser Bin Farhan St, Salah Ad Din, Riyadh 12435, Saudi Arabia

**Keywords:** Big data, Artificial intelligence, Data privacy, Data protection, Health information, Medical device regulation, Healthcare policy

## Abstract

Big data analytics and artificial intelligence are revolutionizing the global healthcare industry. As the world accumulates unfathomable volumes of data and health technology grows more and more critical to the advancement of medicine, policymakers and regulators are faced with tough challenges around data security and data privacy. This paper reviews existing regulatory frameworks for artificial intelligence-based medical devices and health data privacy in Bangladesh. The study is legal research employing a comparative approach where data is collected from primary and secondary legal materials and filtered based on policies relating to medical data privacy and medical device regulation of Bangladesh. Such policies are then compared with benchmark policies of the European Union and the USA to test the adequacy of the present regulatory framework of Bangladesh and identify the gaps in the current regulation. The study highlights the gaps in policy and regulation in Bangladesh that are hampering the widespread adoption of big data analytics and artificial intelligence in the industry. Despite the vast benefits that big data would bring to Bangladesh's healthcare industry, it lacks the proper data governance and legal framework necessary to gain consumer trust and move forward. Policymakers and regulators must work collaboratively with clinicians, patients and industry to adopt a new regulatory framework that harnesses the potential of big data but ensures adequate privacy and security of personal data. The article opens valuable insight to regulators, academicians, researchers and legal practitioners regarding the present regulatory loopholes in Bangladesh involving exploiting the promise of big data in the medical field. The study concludes with the recommendation for future research into the area of privacy as it relates to artificial intelligence-based medical devices should consult the patients' perspective by employing quantitative analysis research methodology.

## 1 Introduction

### 1.1 Background

The use of big data in the healthcare industry is growing across the globe, and its potentiality and benefits are undeniable. Big data analytics, predictive analytics, artificial intelligence or algorithms, machine learning and deep learning can harness large volume datasets. These datasets can be used to improve diagnosis, inform preventative medicine practices and reduce adverse effects of drugs and other treatments. The impact of big data is visible across a variety of clinical settings and fields, including intensive care (Carra et al., 2020), emergency departments, cardiovascular diseases (Leopold et al., 2020), mental health (Simon, 2019), oncology (Patel et al., 2018), paediatrics (Li et al., 2020), psychiatry (Weissman, 2020), preventive care (Batarseh et al., 2020), ophthalmology (Brown et al., 2018), dementia (Ienca et al., 2018), diabetes (Musacchio et al., 2020) and asthma (Tang et al., 2020). In the opinion of Edwards and Veale, as quoted by Professor Mitrou “algorithms increasingly regulate our lives, as they enable or support decisions that they are vital of our welfare and freedoms” (Mitrou, 2019). There are several examples in the literature that discuss the analysis of large quantities of health data, using machine learning technology to diagnose and treat patients (Park et al., 2020).

Bangladesh is only just starting to recognize the potential of big data to make health services more efficient and sustainable. It is not easy for a large developing country, like Bangladesh, to overcome the complex challenges of realizing big data's full potentiality. Nevertheless, the importance of big data is acknowledged by governments. In 2019, Mustafa Jabbar, the minister for posts, telecoms and IT of Bangladesh at the “International Conference on Big Data for Health” said, “we will not be lagging behind in technology. Big data would not be a big challenge for us” (UNDP, 2019). The government's vision of ‘Digital Bangladesh’ capitalizes on fostering innovation and economic growth throughout the country by 2021. Several companies have already offered their services for big data analytics in Bangladesh. It is no secret that big data and predictive analytics would bring positive change into Bangladesh's healthcare industry; however, effectuating such opportunities in real-world settings is a daunting task. It requires new frameworks around the availability and quality of data, data storage, and access to appropriate technological tools for analyzing large volumes of complex, heterogeneous data.

Big data analytics is not all about opportunities. Rather, several issues come into play around data privacy, intellectual property, data security, data ownership, data stewardship and proper data governance. These important ethical and legal issues require an appropriate policy framework.

### 1.2 Research questions and objectives

This paper considers two issues. Firstly, the status of big data analytics in the medical field with a specific focus on whether big data analytics is regarded as a medical device under the current regulatory framework. Secondly, the issue of health data privacy over the use of big data analytics. These two issues inform research questions asked in this paper: Why is current medical device regulation in Bangladesh inadequate to regulate big data analytics-based medical devices? Why is the piecemeal legislation of Bangladesh ineffective for ensuring the security and privacy of health data in the wake of the burgeoning application of big data analytics in the healthcare industry?

To address these questions, this study considers issues affecting the healthcare industry due to the adoption of big data analytics, including present trends and development in healthcare, future prospective, market environment, government policies and regulation and the challenges. The primary objectives are to review the sustainability of the current regulatory framework in Bangladesh, identify gaps and make recommendations for improvements in the context of widespread adoption of big data analytics for healthcare.

### 1.3 Significance of the study

This study illustrates approaches to data privacy law and medical device regulation in Bangladesh and beyond to provide appropriate protections as the healthcare industry seeks to harness the potential of big data while respecting and ensuring the privacy and security of individuals. There is a valuable insight for regulators, academicians, researchers and legal practitioners regarding regulatory loopholes for harnessing the promise of big data in the medical field in Bangladesh. For the purpose of the paper, big data analytics, predictive analytics, artificial intelligence, machine learning, and deep learning will be used interchangeably.

The study apportioned into six sections. Section one discusses the background, research questions, objectives and significance of the study; sections two, three and four address the related work, the methodology, and the study result; section five includes the current stance and governance around big data analytics and artificial intelligence in healthcare and the present regulatory framework of Bangladesh to access the adequacy of the existing policies and regulations to address the research questions of the study. The paper concluded with the policy recommendation. The take-home message for this paper is that although big data would bring novel opportunities to Bangladesh's health industry, it lacks the proper data governance and legal framework necessary for moving forward.

## 2 Related work

### 2.1 Big data analytics

There is no universally acclaimed definition of big data. Generally, big data refers to large volumes of data that traditional software or computational systems cannot process or analyze. Big data may include structured, semi-structured or unstructured data. In addition to large volumes of data, big data encompasses the speed, value, heterogeneity, quality and variety of data. According to Doug Laney, the most popular definition of big data is that it represents three dimensions known as the 3Vs: Volume, Velocity, and Variety (Laney, 2001). The Gartner IT Glossary states, “big data is high volume, high-velocity and/or high-variety information assets that demand cost-effective, innovative forms of information processing that enhance insight, decision making, and processing automation” (Glossary, 2019). Although the 3Vs have been well-accepted for several years, other authors have added the features of veracity, value and variability (Andreu Perez et al., 2015; De Mauro et al., 2016). Over the last few years, more dimensions have been added to the definition of big data as unique (Khan et al., 2019b) (Farooqi et al., 2019) (Shafer, 2017). It is important to note that conceiving each dimension of big data is entirely domain and application dependant. Each dimension represents a specific characteristic of big data that every organization must understand and address to materialize its chosen initiatives.

After analyzing various literature related to big data, De Mauro et al. proposed a formal definition of big data, “Big data is the information asset characterized by such High Volume, Velocity, and Variety to require specific Technological and Analytical Methods for its transformation into value” (De Mauro et al., 2016). Therefore it can be said that big data analytics is the process of accumulating a large volume of heterogeneous, good quality data through technological means and modelling, processing, interpreting, analyzing and validating it to extract meaningful information. Big data analytics can be divided into three categories: descriptive, predictive and prescriptive (Greasley, 2019). These are discussed below:

A. **Descriptive Analytics:** collecting historical data to answer and identify trends in existing processes and prepare that data for further analysis. Descriptive analytics is the foundation for quantifying events, reporting and qualifying big data for actionable insight. Some examples of where this type of analytics is used in healthcare, may be for managing population health by estimating the numbers of particular patients in an area, or identifying areas for improvement in care.
B. **Predictive Analytics:** Predictive analytics is used for predicting and understanding what could happen in the future. It analyzes past data patterns and trends to forecast possible future outcomes. This type of analytics can be used to predict outbreaks and epidemics, identify communities or individuals in need of health services, or predict load default or forecasting weather. The Chicago Department of Public Health used these analytics to develop microtargeting campaigns for mammography screening (Bakker et al., 2020).
C. **Prescriptive Analytics:** Prescriptive analytics help to undertake the best possible course of action based on information learned through descriptive and predictive analysis. It is the most advanced type of analytics, which goes beyond forecasting future events as it can inform the best possible solutions for preventive interventions to avoid risks.

### 2.2 Big data and predictive analytics in healthcare

There is no single definition for big data in healthcare because a one-size-fits-all approach would be “too abstract to be useful” (Auffray et al., 2016). Auffray recommends developing a workable definition that covers high volume, high diversity biological, clinical, lifestyle and environmental information that can only be harnessed through a strong governance model and best practices of new technologies such as advanced analytics (Auffray et al., 2016). Bakker et al. expressed that the terms “advanced”, “best practice”, or “traditional” are time-dependent. Therefore, the definition of big data should encompass a list of analytics that are regarded as advanced or best practice, which is time dependant and updated regularly (Bakker et al., 2020). Big data in healthcare needs to be assessed, updated, and interpreted periodically to ensure maximum output.

**Figure 1:**
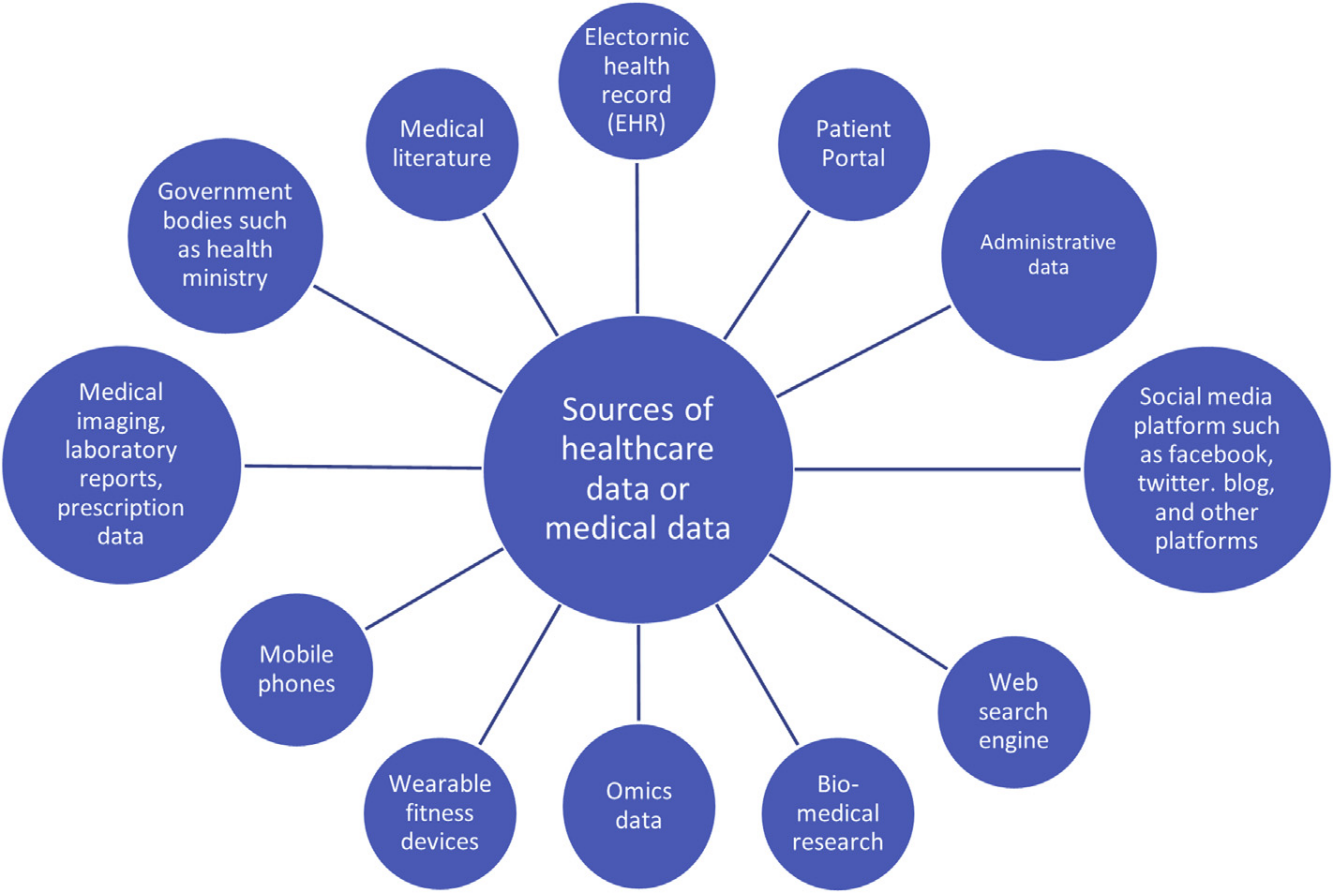
Possible Sources of big data in the healthcare industry.

Big data analytics in healthcare and medicine cover the collection and analysis of large amounts of data from many sources. These sources can include electronic health records, machine, and sensor-generated data, physician notes, prescriptions, medical imaging, laboratory reports, biomedical research, omics data (genomics, transcriptomics, epigenomics, proteomics, metabolomics, interactomics, pharmacogenomics, disease omics) (Ristevski and Chen, 2018), mobile health, social medical platforms, medical literature among others. Some of the sources are illustrated in Figure 1.

The vast pool of medical data does not adhere to any pre-defined model, which means it may be unstructured or semi-structured (Dash et al., 2019). Data integration from various sources makes it difficult to capture or store the data in any specific defined model. Due to the heterogeneous formats and the sheer volume of medical data, it is impossible to process or analyze these using traditional medical technology and software. Artificial intelligence and sophisticated hardware and software such as algorithms or neural networks are necessary to analyze and gain productive insights from medical big data, which can be used for prediction and medical recommendation (Price and Cohen, 2019).

### 2.3 Artificial intelligence as medical device software

The scale and pace of modern technological development have outstripped the existing and longstanding regulatory framework for medical devices designed to regulate traditional medical devices and software. The current regulation was developed based on an extensive and share understanding of related stakeholders (Turpin et al., 2020). The threat of artificial intelligence-based medical software has been recognized worldwide, and regulators from several countries have already proposed frameworks to ensure the safety and effectiveness of artificial intelligence-based software as medical devices (Larson et al., 2021). The need for regulating artificial intelligence-based medical software emanates from the potential pitfalls and implication of this technology (Topol, 2019). International Medical Device Regulators Forum (IMDRF) of the USA, voluntary groups of device regulators, proposed a major regulatory framework to ensure safety and effectiveness of algorithmic-based medical software (IMDRF SaMD Working Group, 2013; 2014; 2015; 2017; Medical Device Clinical Evaluation Working Group, 2019). Besides, the Federal Drug Administration (FDA) of the USA has also issued a discussion paper to address the same (FDA, 2019). Larson et al. opined that these regulations provide a solid foundation for regulating artificial intelligence-based medical software, still lacking comprehensiveness to put complete trust in algorithms (Larson et al., 2021).

Further, the lack of a clear regulatory pathway in the USA and EU for approval and validation of artificial intelligence-based medical devices or algorithms has also undermined these devices' efficacy, quality, and safety (Benjamens et al., 2020; Muehlematter et al., 2021). Therefore, artificial intelligence-based medical devices' potential implications and benefits are still debatable (Stead, 2018). Rivera et al. suggested that regulator should employ regulatory science while developing a new policy framework concerning emerging technologies in the medical field (Cruz Rivera et al., 2021). Regulatory science in healthcare can be defined as “the application of biological, medical and sociological sciences to enhance the development of medicines and devices in order to meet the appropriate standards, quality, safety, and efficacy” (Calvert et al., 2021). Regulatory frameworks encounter parallel development due to integrating evolving medical devices, personalized medicine, big data, and artificial intelligence, which eventually beg the question of their effectiveness (NHSX, 2019).

### 2.4 Health data privacy

The future application of artificial intelligence in medicine entangled with several pressing concerns, for instances, readiness and validation (Wang et al., 2020), Block box algorithm (Bathaee, 2020), biasness (Tschider, 2020), privacy and security of health data (Agrawal and Prabakaran, 2020; Auffray et al., 2016; Chico, 2018; Ching et al., 2018; Price and Cohen, 2019). Furthermore, algorithms have been enabled by the use of labelled big data (Topol, 2019). Hence, protecting the privacy and security of health data cast doubt over the future of artificial intelligence in medicine. The requirement of providing continuous high-quality datasets demands a balance between protecting health data privacy and making data available to improve health and healthcare (McGraw and Mandl, 2021). Such balance is generally guaranteed by the data protection legislation such as Health Insurance Portability and Accountability Act 1996 (HIPAA) of the USA and General Data Protection Regulation 2016 (GDPR) of the European Union (EU). The effectiveness of these legislation in the environment of big data analytics is still debatable (Casey et al., 2019; Gil González and de Hert, 2019; Hoeren and Niehoff, 2018; Kesa and Kerikmae, 2020; McGraw and Mandl, 2021; Turpin et al., 2020). Despite these concerns, artificial intelligence technologies have already shown outstanding results in medicine and have expected to bring further innovations into medical technologies and future healthcare (Park et al., 2020). The future of big data health privacy will be sensitive to data sources, types of data and data custodian (Price and Cohen, 2019). Therefore, it is crucial to have a regulation or regulatory framework that would strike a balance between the significant demand for data and ensure the privacy of health data.

## 3 Methodology

**Figure 2:**
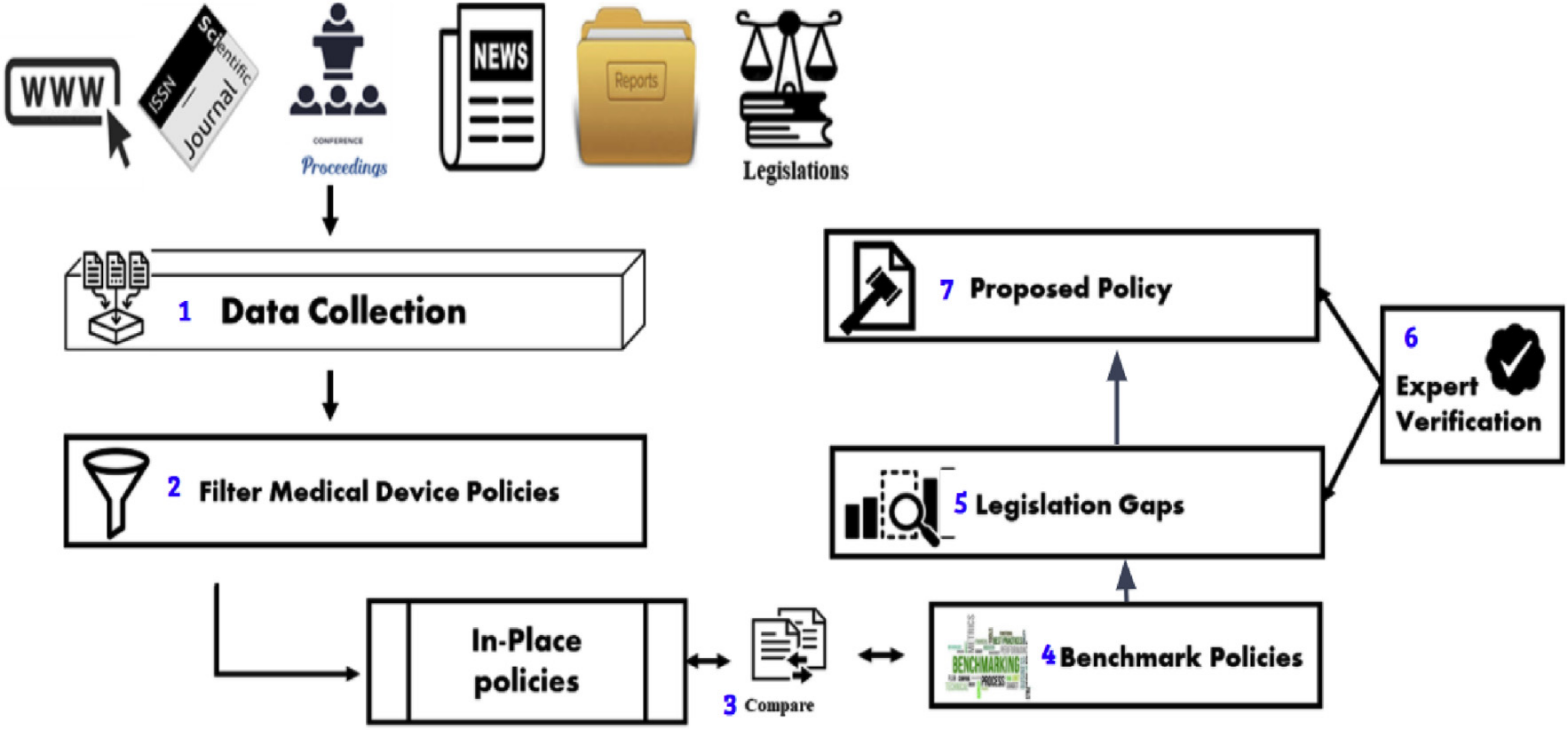
Proposed methodology for creating recommended policies.

This research followed a seven-step approach. The first was the collection of data from primary and secondary legal materials, including statutes, case reports, by-laws, administrative regulations, and browsing through websites, well-respected journals, news and conferences related to selected keywords that are listed in Figure 2. The second step was to filter this data based on policies related to medical data privacy and medical device regulation. These policies were then sorted according to criteria where preferences were set to policies, followed by government reports, journal proceedings and other selected sources. A set of drafts were created as in-place policies that contained all collected policies based on the applied criteria and filters. In-place policies were then compared to benchmark policies collected from respective legislation and policy papers of the European Union and the USA. The comparison demonstrated a gap between effective regulation and that of Bangladesh law on medical device usage based on big data analytics and health data privacy. This gap was verified by Bangladesh legislation and law experts to remove false positives. Finally, a proposed set of guidelines were designed, based on benchmark policies and expert verified gaps. This proposed policy was shared with legislation and law experts for their opinion and to identify conflicting policies. Recommendations were made, based on these research findings, outlining a framework for medical device regulation and health data privacy. There was a critical analysis of the provisions of different legislation to assess the adequacy of these statutes and the identification of loopholes and lacunas in regulations that need immediate attention of the policymakers.

Primary data sources are referred to in the Drugs Act 1940, the Drugs (control) Ordinance 1982, Registration Guidelines for Medical Devices Bangladesh 2015, Digital Security Act 2018, Information and Communication Technology (ICT) Act 2006, Right to Information Act 2009, Medical and Dental Council Act 2010, The Medical Practice Private Clinics and Laboratories (Regulation) (Amendment) Ordinance 1984, the Bangladesh Homeopathic Practitioner Ordinance 1983, Transplantation of Human Organ Act 2018, Community Trust Act 2018, Mental Health Act 2018, Safe Blood Transfusion Law 2002. Secondary material encompassed journals article, law reviews, blogs, reports from international organizations, white papers and other online resources. Secondary data work as a catalyst to discuss, explain, analyze the law to ascertain the implication and scope of the law. The interpretation of law basically come from the judges and scholars of law. Therefore, secondary data in legal analysis plays an integral role. Secondary data may illustrate the current views on how national courts interpret primary data. Sometimes secondary data are so authoritative that even national courts rely on them in their ruling; for instance, the first seminal article, ‘The Right Privacy’ by Warren and Brandies (Warren and Brandeis, 1890), helps develop privacy law in the USA.

## 4 Results

The regulatory framework of Bangladesh for regulating medical devices is ill-equipped to deal with artificial intelligence-based medical devices as it was adopted to regulate hardware-based medical devices and conventional medical software. Unlike traditional medical devices, artificial intelligence-based medical devices expose considerable variance between performance setting environment and real-life practice setting (i.e. technology changes in near real-time based on responses to real-world performance) due to the complexity of the system. Therefore, the current regulatory framework of Bangladesh for medical device regulation is inadequate to regulate artificial intelligence-based medical devices.

On the other hand, health data privacy issues are generally addressed by either comprehensive data protection regulation (EU GDPR) or specific sectoral legislation (The Health Insurance Portability and Accountability Act (HIPAA) Privacy Rules of USA). Presently, Bangladesh has neither comprehensive data protection legislation nor specific sectoral law for health data privacy. The current protection mechanism of different provisions scattered in different legislation is not adequate to protect health data privacy. These provisions were not designed to regulate data governance. Data governance ensures the availability, usability, integrity and security of the data of a particular organization, which are deficit areas in the current regulatory framework of Bangladesh. Therefore, regulators in Bangladesh should seriously ponder these challenges and adopt new regulations.

## 5 Discussion

### 5.1 Bangladesh's stance and governance around big data analytics and artificial intelligence in healthcare

Presently, the principal goal of the Bangladesh government is to exploit information and communication technology (ICT) to address healthcare challenges using digital solutions. This is in line with the strategy and policy focus of Digital Bangladesh Vision 2021. However, several areas need attention before improvements can be made to this effect. Firstly, Bangladesh's Health Information System (HIS) is not fully centralized. Public and private sector healthcare providers keep their data completely separate. Even data generated from public healthcare facilities from urban and rural areas are handled separately. To remove this fragmented record-keeping system in Bangladesh's public healthcare sector, the government created a national data warehouse deploying open-source District Health Information Software 2 (DHIS2) in 2009 (Khan et al., 2019a). DHIS2 is a web-based system designed to collect, validate, analyze and present aggregated statistical data and integrate such data into the health information management system. All data generated from community clinics and tertiary level health facilities and programs are channelled to the health management information system through the software (Management information system, 2018). DHIS2 prioritized data standardization and interoperability of eHealth software and database development. The Management Information System unit (MIS) of the Directorate General of Health Service (MIS-DGHS) of Bangladesh is primarily responsible for catalyzing the health information system through DHIS2.

The National Data Warehouse was developed by the Ministry of Health and Family Welfare (MoHFW) using DHIS2. DHIS2 is now limited to public health service facilities, but the government has been taking initiatives to bring private healthcare facilities and non-governmental organizations to establish a single health data repository system. MIS-DGHS has already developed a draft guideline for “Health Informatics Standards and Data Structure for Bangladesh”. The draft guidelines have set common standards and interoperability procedures applicable to all public, private, non-government and other associated agencies intending to centralize and unify health information systems and allow the seamless exchange of data (Khan et al., 2019a).

Besides, MIS-DGHS has been developing a shared health record (SHR) system that stores lifetime electronic medical records (EMR) for every citizen. Other healthcare facilities can access EMR through the Open Health Information Exchange gateway. Presently, SHR has been used at Kaligonj Upazila Health Complex, Gazipur and Gazipur General Hospital. Furthermore, several public hospitals have already been digitalized, such as Dhaka Medical College and the National Institute of Neuroscience & Hospital (NINS), with many others following suit. Online health bulletin, dashboard, human resource information system (HRIS), health facility registry, telemedicine service, video conferencing, health call centre, social media portal, civil registration and vital statistic (CRVS) have all been implemented to improve healthcare and collect essential statistics. Such statistical data add critical variables to DHIS2, enabling real-time data tracking.

The National Data Warehouse at DGHS will open up new opportunities for the healthcare industry in Bangladesh. Other government agencies like the National Institution of Population Research and Training (NIPORT) (a key institution in providing population research data), the Institution of Epidemiology, Disease Control and Research (IEDCR) (national institution for conducting disease surveillance and outbreak investigation) may provide health routine data as well as surveillance and research data. In the private sector, International Centre for Diarrhoeal Disease Research, Bangladesh (ICCDR,B), Diabetic Association of Bangladesh (BADAS), Bangladesh Institute of Research and Rehabilitation in Diabetes, Endocrine and Metabolic Disorders (BIRDEM), together with large private hospitals such as United Hospital, Evercare Hospital and Square Hospital have already automated their internal systems. Some examples of automation, include patient management, patient records, monitoring availability of essential drugs and blood supply records. Together with potential partners, Bangladesh is now looking to harness massive amounts of data via big data analytics, artificial intelligence, machine learning, deep learning (Khan et al., 2019a). There is no doubt that big data will significantly improve the healthcare system and reduce healthcare costs.

Since January 2019, nearly 70,195 people were reported to have been affected by dengue in Bangladesh. Officially, 57 of those affected were reported dead, and unofficially this number reaches 126 (Dhaka Tribune, 2019). Fortunately, an artificial intelligence tool is being developed by the AIME (Artificial Intelligence in Medical Epidemiology) Inc. that claims to predict dengue outbreaks up to three months in advance (Irwin, 2018). However, Bangladesh has no current policy or strategic guidelines to implement big data in healthcare.

**Figure 3:**
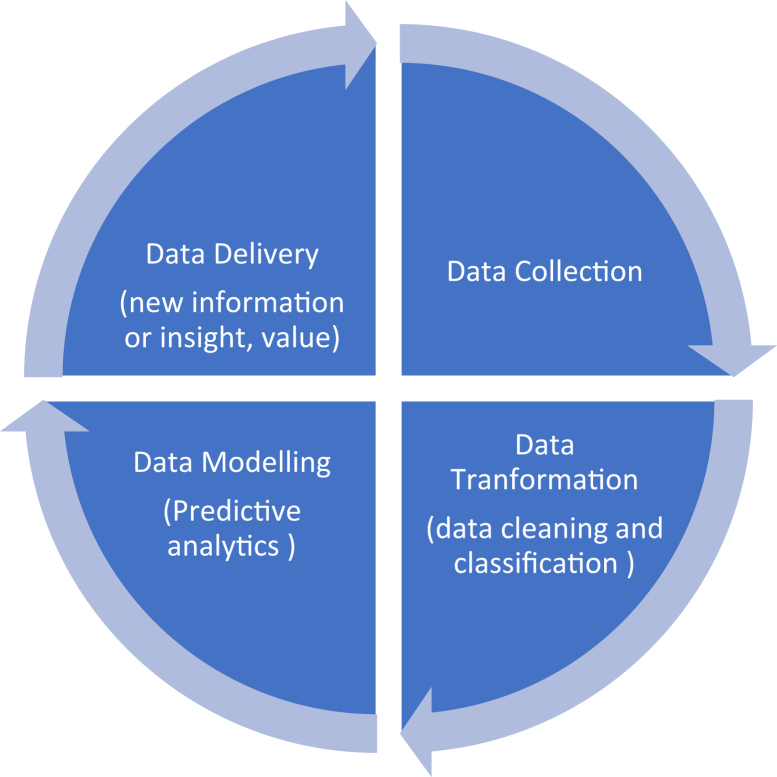
Big data lifecycle in healthcare.

The health industry collects and generates a large pool of heterogeneous data. The underlying objectives and challenges are to extract meaningful insights from these datasets to answer clinical questions, which would not be possible through randomized trials alone (Andreu Perez et al., 2015). It seems natural to mention the need to extract meaningful insight from the data, but big data is a complex system that involves appropriate technical expertise to handle large-scale data. It is a whole new ecosystem that encompasses data collection, data cleansing, data classification, data modelling and data delivery. That ecosystem is known as the big data lifecycle. Data storage, data integrity and access control mechanisms are also part of the big data life cycle. After analyzing different models of the big data lifecycle, Abouelmehdi et al. (2018) summarised the main elements of this lifecycle in healthcare (Figure 3) (Abouelmehdi et al., 2018).

The big data lifecycle requires storage, maintenance, and transmission of a large amount of data to realize maximum benefits for efficient and cost-effective healthcare. As a priority, and at all stages of processing, there should be appropriate technological and organizational measures in place to ensure proper security and privacy of the big data lifecycle. Security of data denotes confidentiality, integrity and data availability, while data privacy refers to appropriate exploitation of data subject information. Lack of proper technical and organizational readiness compromises the security and privacy of users' health data. The issue of data privacy is generally covered by data protection law. Technological measures cover anonymization or encryption of data, data masking, synthetic data, access control mechanism, periodical monitoring and security system auditing. Therefore, before embarking on the big data journey, the government must develop proper policy guidelines for regulating big data analytics-based medical devices and appropriate regulatory frameworks for ensuring the security and privacy of patient's health data.

### 5.2 The current regulatory framework of Bangladesh

Every new invention disrupts the standard workflow because innovation needs implementation and adoption before entering the operation phase. Besides, innovation in the existing environment poses regulatory risks that must be kept in check. Big data analytics or artificial intelligence might cause temporary disruption, but its benefits cannot be overlooked. The application of big data analytics raises several ethical and legal questions. This may explain why big data is not as widespread or fully implemented in the healthcare system as compared to other sectors, like finance and marketing. In Bangladesh, the application of big data, artificial intelligence and machine learning is yet to begin. Thus, one might argue that it is too early to introduce regulation or that early regulation might hamper the flourishment of such technology.

The purpose of regulation should be to protect public interest without disrupting technological innovation while improving the quality and cost-effectiveness of healthcare service. Adverse effects of big data are reflected in a number of recent incidents, for example, the inconsistent performance of big data models for mammogram classification (Wang et al., 2020), and IBM Watson, an artificial intelligence-based medical device, which gave incorrect recommendations on cancer treatments (Strickland, 2019).

The application of predictive analytics or algorithms in healthcare triggers two crucial issues: 1) recognizing artificial intelligence-based medical software under the current regulatory framework and 2) ensuring health data privacy. What's more these analytics are intended to safeguard human lives, which cannot be replaced.

#### 5.2.1 Medical Device Regulation:

Presently, artificial intelligence or machine learning and deep learning are used to augment clinicians' professional services as they have not yet reached full autonomy. Software and hardware to assist medical professionals in their diagnosis or during operations are considered medical devices. However, big data has no value unless analyzed via algorithms. These algorithms may equate to medical software; therefore, big data analytics may also be categorized as medical software. Nonetheless, these classifications around big data remain to be set.

The Drugs Act 1940, the Drugs (Control) Ordinance 1982 and the Drug (Control) (Amendment) Act 2006 regulate the manufacture, import and sale of medical devices in Bangladesh. However, in both legislations, there is no clear definition of what is considered a medical device. Registration Guidelines for Medical Devices Bangladesh 2015 (hereinafter referred to as ‘the Guidelines’) categorize medical devices based on their risk factors (Directorate General of Drug Administration (DGDA), 2015). The purpose of the Guidelines is to ensure standardized and harmonized medical device registration processes and ensure that the Director-General of Drug Administration (DGDA) adheres to the internationally accepted standards of quality, safety and performance regarding medical devices in Bangladesh. According to article 1.1 of the Guidelines, “medical device means any instruments, apparatus, implement, machine, appliance, implant, reagent for in vitro use, software, material or other similar or related article, intended by the manufacturer to be used, alone or in combination, for one or more of the specific medical purpose(s)”.

In a clinical setting, the software is already regarded as a medical device, whether used for clinical or administrative purposes. According to the International Medical Device Regulators Forum the term “Software as Medical Device” (SaMD) is defined as software intended to be used for one or more medical purposes that perform these purposes without being part of a hardware medical device” (IMDRF SaMD Working Group, 2013). The definition includes software intended to be used for medical purposes of “diagnosis, prevention, monitoring, treatment, or alleviation of disease” and other purposes (IMDRF SaMD Working Group, 2013).

For the purpose of this paper, the closest analogy to the algorithms is medical software. Although traditional medical device software and artificial intelligence-based medical devices in healthcare are nearly similar, their working patterns are entirely different. So, why does the algorithm generate so much hype? Why do you need separate regulation to govern this technology? Why is artificial intelligence not treated as medical software like other traditional medical software currently operating in the healthcare industry? The most straightforward answer to these questions is that the risks of using algorithms are far greater than those when using current medical software.

The hype over artificial intelligence is real because of its potential to perform highly complex tasks autonomously without any human intervention. Unlike traditional software, predictive analytics comes with a new set of challenges that have not been taken into consideration, until now. Some of these challenges relate to autonomous working capability, the ability to learn continuously, the ability to change results over time after encountering new datasets and the ‘black box’ nature of the technology (Bathaee, 2020). Algorithms are often called the ‘black box’ problem because of the complexity of processing patterns as they become unreadable except their input and output data. How the algorithm develops a certain result is sometimes beyond the understanding of human beings. In the healthcare context, these challenges have severe implications that require immediate attention.

Artificial intelligence cannot be regulated in the same way as traditional software because of its complex nature and functionality. So, we should not be hasty to lump artificial intelligence or algorithms into the same category as existing medical software without considering the associated risks. Recently, the UK's National Institution for Health and Care Excellence (NICE), in corroboration with National Health Service (NHS) England, Public Health England, and Medcity, published the Evidence Standards Framework for Digital Health Technologies (NICE, 2019). The purpose of this framework is to show “the value of digital health technologies in the UK healthcare system and to establish consistent criteria by which digital health technologies can be assessed”. Further, according to the same document, “Digital Health Technologies comprise a wide range of products used in the healthcare system including apps, software and online platforms that are intended to benefit patients. They may be standalone or combined with other products such as medical devices or diagnostic tests” (NICE, 2019).

The definition of digital health technology laid out in the document includes artificial intelligence as a medical device. These standards differentiated the algorithms into two types, based on the functioning pattern. The first type is the fixed algorithm (output of which is static), and the second is the adaptive algorithm (output of which changes over time in response to new datasets). The standards outlined by NICE are only applicable to the fixed algorithm. Currently, no standards have been developed for the adaptive algorithm. Therefore, there must be clear definitions and categorizations of algorithms before they can be used in the medical field.

The British Standards Institution (BSI) and Association for Advancement of Medical Instrumentation (AAMI) published a report, which was commissioned by the Medicines and Healthcare products Regulatory Agency (MHRA), regarding international standardization of artificial intelligence solutions and deployment in healthcare (Rowley et al., 2019). The US FDA and other stakeholders also contributed to this report. The report urges the development of a common language between traditional health software and artificial intelligence solutions to remove misconceptions. The report also “recognized that any new terminology standards for artificial intelligence in healthcare should be aligned to current international work under development (e.g. ISO/CD 81001-1, Health software and health IT systems safety of product, effectiveness and security, Part-1: Foundational principles, concepts and terms)”. Furthermore, the categorization of artificial intelligence in healthcare should be based on complexities, impacts and benefits, and clinical solutions (Rowley et al., 2019). Further, the IMDRF proposed four risk categories for SaMD application and defined categorizations based on the information provided by the SaMD application to healthcare decisions and condition severity (critical, serious and non-serious) (IMDRF- SaMD Working Group, 2014).

In the healthcare setting, risks associated with artificial intelligence are multi-dimensional, depending on the types of artificial intelligence being developed and deployed. Specifically, fixed algorithm risks would be different from the dangers posed by the adaptive algorithm. Artificial intelligence with an adaptive algorithm poses the question: How do we regulate software that is continuously learning or changing its output in response to new data datasets? Over time, these kinds of changes might introduce unknown risks, overriding existing risks originally envisioned by developers and regulators. Therefore, the NICE recommends developing a separate standard for artificial intelligence with adaptive algorithms (NICE, 2019). The IMDRF further outlined quality management system principles for SaMD to ensure the safety, effectiveness and performance of SaMD in healthcare (IMDRF SaMD Working Group, 2015). These principles include organizational support (leadership, accountability and governance), SaMD lifecycle support (product planning, risk management, record control, improvement of product, measurement and outsourcing), as well as the realization and use processes (requirements management, design, development, verification and validation, deployment, maintenance and decommissioning) (IMDRF SaMD Working Group, 2015).

Traditional legislation similar to the Guidelines of Bangladesh is not designed to regulate software like artificial intelligence. To fully integrate artificial intelligence in Bangladesh's healthcare system, new guidelines or the amendment of existing guidelines are imperative to build trust and positive culture around using this new technology. Medical tools with self-learning and adaptive nature capability warrant completely new regulatory approaches that would ensure patient safety and improve patient care. The Guidelines classify medical devices in Bangladesh based on risk categories, A, B, C and D–low-moderate risk, moderate-high risk, and high risk. Article 2 of the Guidelines stipulate, “the classification rules are based on the intended use and vulnerability of the human body taking into account of the potential risks associated with the technical design and manufacture of the devices.” As such, artificial intelligence software should be categorized based on the potential risk and intended use. Clinical investigation is necessary to assess the safety, clinical performance and effectiveness of a medical device. In another report, the IMDRF recommended a detailed procedure for clinical effectiveness evaluation in the context of the SaMD application (IMDRF SaMD Working Group, 2017; Medical Device Clinical Evaluation Working Group, 2019). The major concerns over artificial intelligence-based medical devices are safety, performance, transparency and accountability. The current approach of Bangladesh is not comprehensive enough to regulate the complexities of artificial intelligence-based medical devices software.

Considering the new risk factors associated with artificial intelligence-based medical devices, the FDA published a discussion paper on the “Proposed Regulatory Framework for Modifications to Artificial Intelligence/Machine Learning (AI/ML)-Based on Software as Medical Device” (FDA, 2019). The FDA paper was not written as a guideline until they requested feedback from different independent stakeholders. The paper suggested the introduction of a Total Product Lifecycle (TPLC) regulatory approach for artificial intelligence machine-based SaMD. According to the discussion paper, “TPLC approach enables the evaluation and monitoring of a software product from its pre-market development to post-market performance, along with the continued demonstration of the organization's excellence” (FDA, 2019).

The paper also provided four general principles based on the TPLC to balance the benefits against the risks of artificial intelligence-based SaMD. The BSI and AAMI further outlined several recommendations for developing a regulatory framework around the application of artificial intelligence in the healthcare system. These recommendations are listed below (Rowley et al., 2019):

➢Forming an international task force for overseeing artificial intelligence in healthcare;
➢Reviewing the existing standards and identifying opportunities;
➢Preparing a layout for terminology and categorization of artificial intelligence in healthcare;
➢Developing guidance for validation processes;
➢Developing a communication and engagement plan.

The USA and Europe have taken incremental steps in regulating artificial intelligence-based medical devices to advance the digital health market. A study conducted by Muehlmatter et al. showed that around 222 devices in the USA and 240 devices in Europe were approved from 2015–2020 (Muehlematter et al., 2021). The effectiveness of these devices remains to be evaluated in the real-world setting. Presently, there is no accredited system to assess or evaluate artificial intelligence-based medical devices’ safety and efficacy after approval. Gerke et al. expressed their concern over the widespread approval and use of artificial intelligence-based medical devices without a full safety assessment and monitoring system (Gerke et al., 2020). Even though the USA and Europe have separate regulation for evaluating artificial intelligence-based medical devices before approval, there is still no specific regulatory pathway (Muehlematter et al., 2021). Unlike traditional drugs and medical devices or software, artificial intelligence-based SaMD would show noticeable variance between their performance in the testing environment and actual practice settings due to their working system (Gerke et al., 2020). Therefore, they would probably pose more risks than potential benefits. Larson et al. identified significant shortcomings in USA and Europe regulatory frameworks for artificial intelligence-based medical devices related to the lack of resources to assess performance, insufficient characterization of safety and performance elements, and inherent conflicts of interest (Larson et al., 2021).

A regulatory framework for artificial intelligence-based SaMD must be aligned with the present regulatory framework. However, Bangladesh has not yet fully integrated artificial intelligence into its healthcare system, and regulatory approaches are needed to gain trust from healthcare professionals and patients before deploying artificial intelligence solutions. Although artificial intelligence in healthcare could be considered SaMD under the current Guidelines, these Guidelines are insufficient to regulate artificial intelligence-based medical devices. FDA acknowledged that the current regulatory framework for traditional medical devices is not suitable for artificial intelligence-based SaMDs due to their rapid development and modifications (Park et al., 2020).

The foregoing discussion identified that there are several reasons that traditional medical device regulation is not suitable for regulating artificial intelligence-based medical devices. Firstly, though the algorithm can be equated to the traditional medical device software, there is a considerable difference in their functioning patterns. Artificial intelligence-based medical devices are not only run autonomously but also capable of making clinical decisions on behalf of doctors. Unlike traditional medical device software, artificial intelligence-based medical devices are continuously evolving and updating with new datasets. Secondly, the lack of transparency, explainability, accountability and predictability on the part of algorithmic devices could pose unintended side effects to patients. The artificial intelligence-based medical device might be biased or discriminatory due to lack of quality data. The decisions made by the artificial intelligence system may not be universally acceptable. Even if the inference drawn by the algorithm is correct, the result might not be accepted due to the complexity of explaining the reasons behind making such inferences. Thirdly, effective implementation of artificial intelligence-based medical devices into the healthcare system requires a whole new organizational and technical infrastructure to ensure patient safety, reduce health system risk, establish trust and facilitate wide adoption. Implementing artificial intelligence-based medical devices into the current healthcare system requires ensuring continuous availability of high-quality data, data standardization and integration into existing clinical workflow, interoperable data sharing technology and establishing appropriate cybersecurity measures. Fourthly, the lack of appropriate artificial intelligence policy and regulatory frameworks to develop proper transparency, accountability and liability regime.

It is high time that legislators of Bangladesh should address issues around the application of big data analytics or artificial intelligence in the healthcare industry. Apart from developing artificial intelligence algorithms, the productization process necessitates data sharing, transparency, patient safety, data standardization and integration into the existing workflow, and proper education in artificial intelligence (He et al., 2019). Therefore, legislators must develop new guidelines with appropriate precertification and post-market oversights to regulate artificial intelligence-based medical devices. In addition, Bangladesh's medical and dental council should develop clear guidance and codes of conduct on the appropriate use of big data and artificial intelligence by health professionals.

#### 5.2.2 Data Protection and Privacy

The sensitivity of health data is well understood; as quoted by Kaplan on the work of Beyleveld and Histed regarding confidentiality and sensitiveness of health data:

“Information that patients provide for their treatment is about very personal and sensitive areas of their lives. Indeed, it relates to their very existence, both physically and symbolically. As such, it is not information that they may be presumed to be prepared to disclose or have used freely. It is their vulnerability, constituted by pain and distress, or fears about their health and lives, that leads them to disclose this information to health professionals. At the same time, people are apt to attach great importance to intimate information about themselves and their bodies, and this can be associated with mystical and religious belief, which by their nature can be idiosyncratic.” (Kaplan, 2016).

**Figure 4:**
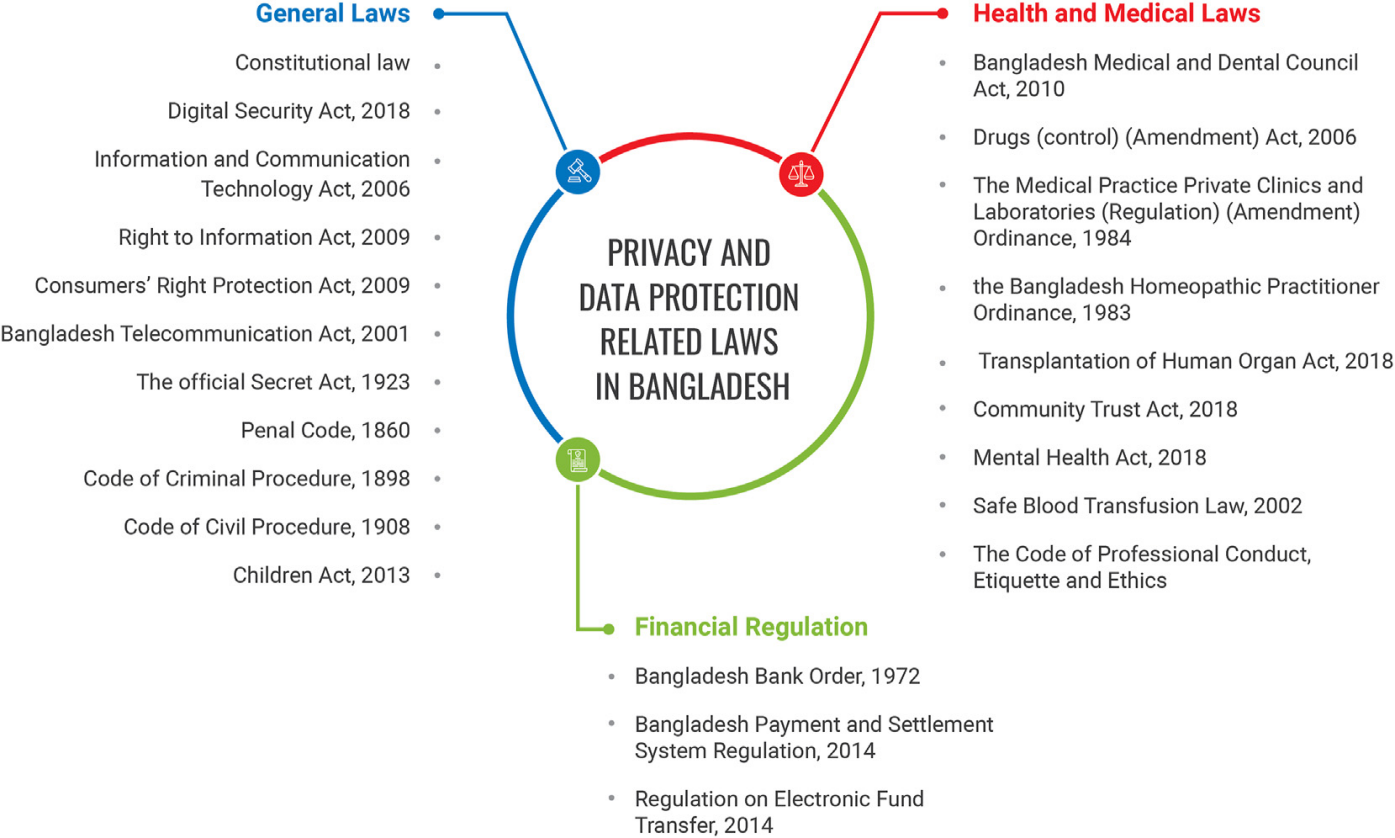
General overview of privacy and data protection laws in Bangladesh.

Big data in the medical industry brings with it a complex array of legal and ethical concerns relating to privacy, reliability, safety and liability. Every data protection legislation around the world requires stringent security when it comes to individual health and medical data. In general, the threat against data privacy has intensified since the technological revolution, and big data has aggravated this threat even further. Concerning health data, the threats of big data could be catastrophic. This is not to say that every big data application poses a privacy threat. For example, there are applications where data collection and processing would not be classified as personal (Sartore, 2016). In the opinion of one author, “the big data age is not only levering the degree of risk its application can generate, the new threat would be the same in nature but higher in intensity” (Sartore, 2016). There are several general and sector-specific laws in Bangladesh, as specified in Figure 4, that expressly and impliedly ensure data privacy. The present study discusses only those laws that are relevant to the purpose of the study.

Data protection legislation generally addresses data privacy issues. However, presently Bangladesh does not have comprehensive data protection law. Thus it is pertinent to access existing sectoral legislation to test sustainability in the age of big data, artificial intelligence, machine learning, and deep learning. The closest provision dealing with data privacy in Bangladesh can be found in section 26 of the Digital Security Act (DSA) 2018. The provision criminalizes the use of identity information without permission. Stating that if any person without any legal authority collects, sells, takes possession, supplies or uses any person's identity information, then that will be an offence.

The explanatory note of section 26 defines “identity information” as any external, biological or physical information or any other information which singly or jointly can identify a person or a system, his/her name, address, date of birth, parent's name, signature, national identity number, birth or death registration number, driver's license, E-Tin, electronic or digital signature, username, credit or debit card number, voice print, retina or iris image, DNA profile, security-related questions or any other identification. Notably, this definition also covers the health or medical information of an individual.

The major problem with section 26 is that it is reactive in nature, which is different from comprehensive data protection legislation. One of the core ideas of comprehensive data protection legislation is to empower citizenry with self-determination and give control to the citizenry over their personal data. The foundation of data protection legislation relies on data protection principles such as collection limitation, purpose specification, data minimization, data integrity, security and access. The notion of these principles reflects proactiveness, meaning that individuals should know how their personal information will be used before they share it.

Careful analysis of section 26 of the DSA reveals it can, to some extent, ensure data privacy, but it will be ineffective in many circumstances. The provision speaks more to misusing identity information without legal authority. The term “without legal authority” should be explained by the DSA for the purpose of this provision because data privacy can still be compromised with legal authority. Hypothetically, if personally identifiable information is collected with the consent of concerned individuals for healthcare purposes, this data may be used for another purpose. In such instances section 26 is of no avail. This can occur because the individual has no idea how the data will be used as there is no provision of notice or choice. Also, there is no transparency and accountability on the part of the data collector. So, there is no scope of knowing how personal information will be used and how long it will be kept. Section 26 is a big step towards realizing the importance of personal data protection for the Bangladesh legal system; however, it is still a far cry from ensuring personal data privacy, particularly in the context of big data.

The DSA could also be useful in ensuring digital infrastructure security, for instance, computers, digital devices or digital networks. The DSA criminalizes unauthorized access (Section 18), damaging of computer or computer system (Section 19), change of computer source code (Section 20), digital or electronic forgery (Section 22), digital or electronic fraud (Section 23), identity fraud (Section 24), hacking (Section 34), and online defamation (Section 29). These provisions, to some extent, ensure confidentiality, integrity and security of personal information. Nonetheless, in the age of big data, the collection of data is growing exponentially and maintaining confidentially, integrity, security and privacy is a daunting task.

As mentioned earlier, the risk of compromising health data is far greater than other forms of data necessitating a higher level of protection. Section 15 of the DSA could provide extra protection; it stipulates that the government has the authority to declare any computer system, network or information infrastructure as a critical information infrastructure. In the absence of any comprehensive data protection law, declaring the national data warehouse or integrated health information system as critical information infrastructure may be a feasible solution.

On the other hand, section 63 of the Information and Communication Technology (ICT) Act 2006 mandates electronic records' privacy and confidentiality, providing that the word “privacy” is explicitly mentioned. As the heading of section 63 states, “punishment for disclosure of confidentiality and privacy”. Section 63 protects against internal unauthorized disclosure viz. the person who has the authority to have access to any electronic record, book or register but disclose the same without the consent of the individual concerned. This provision could act as a shield against unauthorized disclosure of patients’ medical or health records and prevent their use for personal gain.

Presently, the government is the biggest repository of health data. In the context of government accountability, the Right to Information Act (RTI) 2009 ensures the privacy of health data to some extent. One of the purposes of the RTI Act is to provide the right of access to information by the citizenry held by the government to ensure transparency in its affairs. The preamble of the RTI Act stipulates “all powers of the Republic belong to the people, and it is necessary to ensure the right to information for the empowerment of the people.” As quoted by one author, “it creates a judicially enforceable policy that favours a general philosophy of full disclosure. . . based on democratic political theory and a philosophy of open government” (Hacker and Petkova, 2016). Section 4 of the RTI Act mandates that government agencies are obligated to supply any information as requested by any citizen. Every authority shall preserve or store all information in a manner that ensures the right of access to information by every citizen held by government agencies (Section 5). The authorities are exempted from such obligation on the grounds of national security, personal privacy, trade and commercial secrecy, defence, international relation and interference with a criminal investigation and criminal justice system (Section 7).

The RTI Act can serve the purpose of protecting informational privacy in the context of health data. Authorities are not obliged to respect every request if they deem that publication of requested information might offend or violate personal privacy or endanger the physical safety of any person. In the absence of comprehensive data protection legislation, the RTI Act would be useful to hold the government accountable for misusing public information. One of the main concerns of the RTI Act is that people are unaware of the degree to which their personal information is being collected and stored by the government. So, if people do not know that information exists, how can they ask to view it?

Furthermore, the Snowden revelations beg the question of the effectiveness of the legislation like the RTI Act (Greenwald et al., 2013). The scope of the RTI Act is limited to government authorities. Private entities have free rein to use the personal information of citizenry without having any accountability.

There are also numerous laws in the medical field such as the Bangladesh Medical and Dental Council Act 2010, the Drugs (Control) (Amendment) Act 2006, The Medical Practice Private Clinics and Laboratories (Regulation) (Amendment) Ordinance 1984, the Bangladesh Homeopathic Practitioner Ordinance 1983, Transplantation of Human Organ Act 2018, Community Trust Act 2018, Mental Health Act 2018, Safe Blood Transfusion Law 2002. Surprisingly, none of these legislations has explicitly provided any provision for data privacy. Traditionally, it is the duty of medical practitioners to uphold the confidentiality and privacy of patients’ medical records. The Code of Professional Conduct, Etiquette and Ethics mandate maintaining confidentiality and privacy of patient information (WHO, 2012). Nonetheless, the application of this code is limited to medical professionals. In the present data-driven economy, the importance of data protection is undeniable. The importance is reflected by witnessing the clear surge of adopting comprehensive data protection legislation around the world. Presently, 142 countries have comprehensive data protection law (Greenleaf, 2020). It does not necessarily mean that sectoral legislation is less effective. Sectoral-based legislation with appropriate fair information practice principles can ensure personal data privacy. Unlike Europe, the US does not have comprehensive privacy legislation. Chesterman opined that “across Asia, the absence of European-style rights protection meant that an approach similar to the US model taken, with piecemeal legislation or episode court intervention or, as in many jurisdictions the matter was either left to the market or essentially ignored” (Chik et al., 2014).

Presently, Bangladesh has neither comprehensive data protection legislation nor specific sectoral legislation that explicitly addresses patient data privacy. As analyzed before, the current protection mechanism is primarily based on the different provisions scattered in separate legislation that hardly ensure adequate protection of patient data privacy. Among these legislations, section 26 of the DSA specifically addresses personal data privacy. This section is only effective in the event of using personally identifiable data without legal authority. Now, most of the misuse of personal data happens with legal authority. For example, data users collect the data with proper consent from the data subject and later use it for purposes other than that which legal authority was initially given. Specific sectoral based legislation can be effective in protecting the privacy of medical data. HIPAA, 1996 in the US ensures health data privacy, though the effectiveness of the Act comes into question in the wake of current technological development (McGraw and Mandl, 2021). The efficacy of HIPPA will not be discussed here as it requires a whole study of its own.

The piecemeal legislation of Bangladesh lacks core elements of data protection legislation, such as fair information practice principles, rights of the data subject, obligation of the controller or data users, supervisory authority and enforcement mechanisms. Further, artificial intelligence put privacy protection at the crossroads of balancing between protecting patients’ data and making data available to improve health and healthcare. Therefore, policymakers and regulators in Bangladesh must work to adopt comprehensive privacy and security protections for health data.

Considering the abovementioned legislation, it is high time for Bangladesh to adopt comprehensive data protection legislation. In the age of technological revolution, such sectoral legislation with minimal protection would not be effective in preserving the privacy of peoples' personal information. Apart from the western world, several countries in Asia, including Singapore, Malaysia, Hong Kong and Thailand, have adopted data protection legislation. India has also drafted its Personal Data Protection Bill in line with the recently enacted European Union's General Data Protection Regulation. Following closely behind is Indonesia.

## 6 Conclusion and recommendations

Artificial intelligence–based medical devices or software are different from traditional medical software in terms of learning capacity, ubiquitous in medical interaction, making recommendations, and opaque nature of making such recommendations (the logic behind making recommendations are often opaque to physicians). The conventional medical devices regulation is not fit to regulate such emerging technologies. Therefore, the present legal frameworks of Bangladesh for medical devices do not suffice for artificial intelligence-based medical devices, leading to regulatory uncertainty over the use of these devices. The responsible regulatory authorities should consider adopting a new policy framework that aligns with current international standards to ensure the safe and effective use of artificial intelligence-based medical devices in Bangladesh. Bangladesh regulators should consult the recent FDA discussion paper (FDA, 2019) and the framework, principles and guidance of the IMDRF (IMDRF SaMD Working Group, 2013; 2014; 2015; 2017; Medical Device Clinical Evaluation Working Group, 2019) to establish sustainable and harmonized approaches for regulatory control of artificial intelligence-based medical devices.

Concerning health data privacy, the current protection mechanism in Bangladesh of different provisions scattered across different legislations lacks two fundamental elements of data protection regulation: data governance and enforcement mechanism. To promote and establish an appropriate data privacy regime, importance should be given to equitable and responsible use of health data that fosters health and healthcare systems and ensures patient health data privacy. Policymakers should meaningfully set one governance regime by enacting comprehensive data protection legislation where the European Union's General Data Protection Regulation (GDPR) could be used as a benchmark.

In the context of artificial intelligence, protecting health data while fostering artificial intelligence development will not be easy. Therefore, policymakers should create a balanced framework where patients are assured of their data privacy and the implementation of artificial intelligence to improve the healthcare system. To develop a balanced framework for data protection, regulators need to adopt an interdisciplinary approach because of technical complexities associated with artificial intelligence. The framework should be developed in cooperation with the engineers and computer science experts to ensure that legislation takes complete account of the technical nature of the regulated matter and provides an appropriate safeguard.

A significant limitation of this is the lack of materials and related data around the use of artificial intelligence-based medical devices from Bangladesh's perspective. Although there are plethora of articles on the subject as a whole, there is hardly any writeup around Bangladesh's stance. Few authors focus on big data analytics and less address the issue of regulatory challenges.

It is important to continue following the status of big data and the healthcare industry in Bangladesh to ensure that policy and regulatory frameworks are constantly adapted to the rapidly changing field of artificial intelligence–based medical devices. Without the right policy and regulation, this area of exponential growth may hamper advances in medicine that are critical to better health outcomes. It is our recommendation that future research into the area of privacy as it relates to artificial intelligence-based medical devices should consult the patients’ perspective.

## Declarations

### Author contribution statement

Shafiqul Hassan: Conceived and designed the experiments; Contributed reagents, materials, analysis tools or data; Wrote the paper.

Mohsin Dhali: Conceived and designed the experiments; Contributed reagents, materials, analysis tools or data; Wrote the paper.

Fazluz Zaman: Analyzed and interpreted the data; Contributed reagents, materials, analysis tools or data.

Muhammad Tanveer: Performed the experiments; Contributed reagents, materials, analysis tools or data.

### Funding statement

This work was supported by Governance and Policy Design Research Lab of 10.13039/501100012639Prince Sultan University.

### Data availability statement

Data included in article.

### Declaration of interests statement

The authors declare no conflict of interest.

### Additional information

No additional information is available for this paper.
